# Metabolomic profile of cerebrospinal fluid from patients with diffuse gliomas

**DOI:** 10.1007/s00415-024-12667-9

**Published:** 2024-09-03

**Authors:** Nora Möhn, Harold F. Hounchonou, Sandra Nay, Philipp Schwenkenbecher, Lea Grote-Levi, Fadi Al-Tarawni, Majid Esmaeilzadeh, Sven Schuchardt, Kerstin Schwabe, Herbert Hildebrandt, Hauke Thiesler, Friedrich Feuerhake, Christian Hartmann, Thomas Skripuletz, Joachim K. Krauss

**Affiliations:** 1 Department of Neurology, Hannover Medical School, Carl-Neuberg-Straße 1, 30625 Hannover, Germany; 2 Department of Neurosurgery, Hannover Medical School, Hannover, Germany; 3 Fraunhofer Institute of Toxicology and Experimental Medicine, Hannover, Germany; 4 Institute of Clinical Biochemistry, Hannover Medical School, Hannover, Germany; 5 Department of Neuropathology, Institute of Pathology, Hannover Medical School, Hannover, Germany

**Keywords:** Metabolomic profile, Cerebrospinal fluid, Gliomas, Putrescine, Biomarkers

## Abstract

**Background:**

Diffuse gliomas are among the most common brain tumors in adults and are associated with a dismal prognosis, especially in patients with glioblastoma. To date, tumor tissue acquisition is mandatory for conclusive diagnosis and therapeutic decision-making. In this study, we aimed to identify possible diagnostic and prognostic biomarkers in cerebrospinal fluid (CSF) and blood.

**Methods:**

During glioma surgery at our institution, CSF and blood samples were collected from patients. Subsequently, targeted metabolomics analysis was used to detect and quantify circulating metabolites. The metabolome profiles of glioma patients were compared with those of patients in a control group who had undergone neurosurgery for other entities, such as nonglial tumors or hydrocephalus, and were correlated with established glioma diagnostic molecular markers.

**Results:**

In this study, a total of 30 glioma patients were included, along with a control group of 21 patients without glioma. Serum metabolomic analysis did not detect any significant differences between the groups, whereas CSF-metabolome analysis revealed increased levels of six metabolites in glioma patients. Among these, the most pronounced differences were found for the biogenic amine putrescine (*p* = 0.00005). p-Cresol sulfate was identified as a potential CSF marker for determining isocitrate dehydrogenase (IDH) status in glioma patients (*p* = 0.0037).

**Conclusion:**

CSF-metabolome profiling, unlike blood profiling, shows promise as a diagnostic tool for glioma patients with the potential to assign molecular subtypes. The next step will involve a larger multicenter study to validate these findings, with the ultimate objective of integrating CSF metabolomics analysis into clinical practice.

**Supplementary Information:**

The online version contains supplementary material available at 10.1007/s00415-024-12667-9.

## Background

Gliomas are the most common brain tumors, with an incidence varying from 4.67 to 5.73 per 100,000 persons [1, 2]. They are classified and graded according to the WHO classification of tumors of the central nervous system based on their histological appearance and molecular characteristics [3]. However, the preoperative diagnosis is mainly dependent on neuroimaging. Patient-related prognostic factors include patient age and Karnofsky Performance Score, whereas the most accurate prognostic and diagnostic indicators are tumor-related genetic markers. These include mutations in the isocitrate dehydrogenase (IDH) genes (*IDH1* and *IDH2*), the 1p/19q codeletion, O6-methylguanine methyltransferase (MGMT) promoter methylation, cyclin-dependent kinase inhibitor 2A/B (*CDKN2A/B*) gene homozygous deletion, telomerase reverse transcriptase (*TERT*) promoter mutations, and epidermal growth factor receptor (*EGFR*) alterations, + 7/− 10 chromosome copy-number alterations and H3 p.K28 mutations [3]. To date, tumor tissue examinations are mandatory for the final diagnosis. The established therapeutic options include surgical resection followed by radiotherapy and chemotherapy, mostly with alkylating agents [4]. In cases of unclear lesions or deep-seated tumors, stereotactic biopsy is needed for tissue acquisition and further therapeutic decisions. Considering the currently available multimodal treatment options, the prognosis remains poor, especially in glioblastoma patients, who have an average overall survival of approximately 16 months [5].

In the past decade, the concept of liquid biopsy has gained increased importance in cancer research. Circulating tumor DNA, microRNA, long noncoding RNA, tumor-derived proteins, extracellular vesicles, and tumor cells in blood and cerebrospinal fluid (CSF) have been identified as potential diagnostic and prognostic markers in gliomas, yet this subject has received little attention thus far [6, 7]. Moreover, the topic of metabolomic studies in patients with gliomas remains largely underexplored. While a recent study on the metabolic profile of glioma tumor tissue demonstrated a correlation with the current WHO classification [8], data about circulating metabolites are needed.

In this study, we aimed to identify and quantify circulating metabolites in the blood and CSF of glioma patients by performing targeted metabolomics analysis. Furthermore, we explored the diagnostic value of the detected metabolites by analyzing their potential correlation with established molecular genetic markers.

## Methods

### Study design and patient selection

A collaborative project between the Department of Neurology and the Department of Neurosurgery at Hannover Medical School was initiated to identify new biomarkers in gliomas. After providing written informed consent, patients who required surgery for brain tumor removal were recruited for the study. Patients with nonglial tumors or patients who needed cranial surgery for other reasons (for example, hydrocephalus) served as controls. CSF and serum were collected from the patients intraoperatively. The CSF was taken at the beginning of the operation before the tumor was resected. Afterward, the biomaterial was immediately transferred to the Neurochemistry Laboratory of the Department of Neurology, where it was further processed and then frozen at − 80 °C. The maximum time from sampling to freezing was approximately 1 h. Neuropathological evaluation was performed according to the 2021 “World Health Organization Histological Classification of Tumors of the Central Nervous System” [3]. The study protocol was approved by the Ethics Committee at Hannover Medical School (No. 8269_BO_S_2019).

### Targeted metabolomic analysis

All CSF and serum samples were stored at − 80 °C. For analysis, the samples were shipped to Fraunhofer ITEM (Hannover) on dry ice and analyzed in duplicate using a targeted metabolomics kit (MxP Quant 500 kit, Biocrates Life Science AG, Innsbruck, Austria). The concentrations of metabolites were measured on an AB SCIEX 5500 QTrapTM mass spectrometer (AB SCIEX, Darmstadt, Germany). Metabolite extraction and analyses were conducted following the manufacturer’s protocol (https://biocrates.com/mxp-quant-500-kit, accessed on 05 June 2023).

**Data processing**: After normalization and preprocessing of the data, MetIDQTM software (Biocrates) was used for peak integration and subsequent calculation of metabolite concentrations. All analytes that were above the limit of detection (LOD) in ≥ 80% of patients in at least one group were selected for further investigation as previously described [9, 10]. Values below the LOD were replaced by the reference value provided by the manufacturer (LOD/2). Missing values were replaced by the samplewise *k*-nearest neighbor algorithm using MetaboAnalyst 5.0.

### Statistical analysis

The metabolite concentration data were entered into Metaboanalyst 5.0 software for multivariate analyses, including *t* tests, where Benjamini–Hochberg correction was applied with a false discovery rate (FDR) of 0.1 to account for multiple testing. A fold-change (FC) threshold of > 2 was set for differential abundance analysis, which was visualized as a volcano plot. Classical univariate receiver-operating characteristic (ROC) curve analysis was employed to evaluate the performance of the identified metabolites as possible biomarkers. Differential abundance analysis and receiver-operating characteristic (ROC) curve analysis were performed using the open source tool MetaboAnalyst 5.0 (http://www.metaboanalyst.ca).

The abbreviations used for phosphatidylcholines (PCs), triglycerides (TGs), sphingomyelins (SMs), hexosylceramides (HexCers), and ceramides (Cers) correspond to the Biocrates nomenclature.

Age and BMI are expressed as the median ± SD, and differences between groups were analyzed using the Mann‒Whitney test. Group comparisons for sex and comorbidities were performed with Fisher’s exact test.

## Results

### Cohort description

A total of 30 patients who underwent surgery for gliomas were included in this study. Surgery was performed according to the standard departmental techniques as described previously [11]. In 5 patients (17%), surgery was performed due to tumor recurrence (Table 1). Neuropathological evaluation according to the 2021 “World Health Organization Histological Classification of Tumors of the Central Nervous System” [3] revealed glioblastoma IDH-wildtype (CNS WHO grade 4) in 22 patients, oligodendroglioma IDH-mutant and 1p/19q-codeleted CNS WHO grade 2 in 2 patients and CNS WHO grade 3 in one patient, diffuse astrocytoma IDH-mutant (CNS WHO grade 2) in 2 patients, and astrocytoma IDH-mutant (CNS WHO grade 3), as well as diffuse midline glioma H3 K27-altered (CNS WHO grade 4) in one patient, respectively.

**Table 1:** Clinical presentation, tumor characteristics, and therapy of the glioma cohort

ID	Sex	Age (years)	Tumor	Symptoms	KPS	IDH status	MGMT methylation	Location	Tumor diameter (mm)	Extensive necrosis	Dexamethason (mg/day)	Surgery	Adjuvant therapy
1	M	61	O°2	Seizure	90	Mut	N/A	Temporal / cortical	24	−	−	STB	CRTx
2	M	77	GBM°4	Aphasia	90	WT	No	Temporal / cortical	52	+	24	GTR	RTx
3	M	62	GBM°4	Motor deficit	80	WT	No	Parietal / subcortical	21	−	24	GTR	CRTx + TTF
4	M	64	GBM°4	Motor deficit	50	WT	Yes	Thalamus / subcortical	50	+	−	STR	RTx
5	M	31	A°3 (recurrence)	None	90	Mut	N/A	Frontal / cortical	48	−	−	STR	CRTx
6	F	79	GBM°4	Aphasia	70	WT	No	Temporal / cortical	60	−	16	STR	RTx
7	F	55	GBM°4	Motor deficit	70	WT	Yes	Parietomesencephal / subcortical	47	+	24	STR	RTx
8	F	77	GBM°4	Motor deficit	60	WT	Yes	Frontotemporal / subcortical	50	+	12	STR	n.a
9	F	56	GBM°4 (recurrence)	None	100	WT	N/A	Frontotemporal / cortical	25	+	8	GTR	CTx
10	M	50	GBM°4	Motor deficit / seizure	90	WT	No	Temporal / cortical	14	−	−	GTR	CRTx
11	M	49	DMG°4	Cephalgia	90	WT	N/A	Medulla oblongata / subcortical	26	−	−	Biopsy	RTx
12	F	74	GBM°4	Visual impairment	80	WT	N/A	Occipital / subcortical	52	+	−	GTR	RTx
13	M	64	O°3 (recurrence)	none	50	Mut	N/A	Frontal / cortical	15	−	−	GTR	CTx
14	M	59	O°2	Seizure	90	Mut	N/A	Frontal/cortical	52	−	−	GTR	CRTx
15	M	83	GBM°4 (recurrence)	None	90	WT	No	Temporal/cortical	25	+	−	GTR	RTx
16	M	49	GBM°4 (recurrence)	None	90	Mut	N/A	Frontal/cortical	16	−	−	GTR	RTx
17	F	66	GBM°4	Seizure	70	WT	Yes	Temporal/subcortical	70	+	−	GTR	BSC
18	M	59	GBM°4	Incidental finding	90	WT	Yes	Temporal/cortical	40	+	−	STB	CTx
19	F	21	A°2	Seizure	90	Mut	Yes	Parietal/cortical	32	−	−	STR	None
20	M	61	GBM°4	Aphasia + visual impairment	80	WT	Yes	Temporal/subcortical	65	+	16	GTR	CRTx
21	M	82	GBM°4	Motor deficit	80	WT	No	Frontal/cortical	46	+	24	GTR	RTx
22	M	83	GBM°4	Adynamia	60	WT	No	Frontal/cortical	57	+	32	STR	BSC
23	M	74	GBM°4	Mental confusion	70	WT	No	Parietal/subcortical	51	+	12	STR	RTx
24	F	31	A°2	Cephalgia + vertigo	90	Mut	N/A	Frontal/cortical	20	−	−	GTR	None
25	M	58	GBM°4	Motor deficit	50	WT	Yes	Temporal/cortical	25	+	16	GTR	BSC
26	M	38	CNS HGNETBCOR	Seizure	90	WT	Yes	Parietal/cortical	20	−	24	STR	CTx
27	M	56	GBM°4	Mental confusion	80	WT	No	Corpus callosum/subcortical	59	+	12	STR	RTx
28	M	80	GBM°4	Seizure	80	WT	No	Frontal/cortical	38	+	24	GTR	CRTx
29	F	71	GBM°4	Aphasia	70	WT	No	Frontal/subcortical	48	+	12	STR	n.a
30	F	80	GBM°4	Motor deficit	40	WT	No	Frontotemporal/cortical	72	+	24	GTR	n.a

*M* male, *F* female, *A°2/°3* astrocytoma CNS WHO grade 2/grade, *CNS HGNET-BCOR* neuroepithelial tumor with EP300::BCOR(L1) fusion, *DMG°4* diffuse midline glioma CNS WHO grade 4, *GBM°4* glioblastoma CNS WHO grade 4, *O°2/°3* oligodendroglioma CNS WHO grade 2/grade 3, *KPS* Karnofsky Performance Score, *IDH* Isocitrate dehydrogenase, *MGMT* O6-methylguanine methyltransferase, *WT* wild type, *Mut* mutant, *STB* stereotactic biopsy, *GTR* gross total resection, *STR* subtotal resection, *CRTx* chemoradiotherapy, *RTx* radiotherapy, *CTx* chemotherapy, *BSC* best supportive care, *TTF* tumor treating fields, *N/A* not applicable

One patient showed a diffuse glioma without morphological signs of malignancy. The tumor exhibited no *IDH1*, *IDH2*, *TERT*, H3, or *BRAF* mutation, and had no *EGFR* amplification, no *MYB* rearrangement, and a positive *MGMT* status. The epigenetic profile of the tumor corresponded to the methylation class ‘neuroepithelial tumor with EP300:BCOR(L1) fusion’ in the Heidelberg brain tumor classifier version 12.3 (www.molecularneuropathology.org/mnp/classifiers/10). IDH1/2 mutations were found in 7 patients. The *MGMT* gene promoter status was analyzed in 19 patients with GBM, one with neuroepithelial tumor with EP300::BCOR(L1) fusion, and one with astrocytoma CNS WHO grade 2. Hypermethylation was detected in nine patients. Table 1 displays the clinical presentation, tumor characteristics, and treatment of the patients in the glioma group. In one patient (No. 30, Table 1), no CSF samples were available.

The control group consisted of 21 patients with no glioma. The detailed patient characteristics are provided in the supplementary material (Table S1). The patients’ ages ranged between 21 and 83 years in the glioma group and between 17 and 82 years in the control group (median age: glioma: 61.5 years; control: 56 years; *p* = 0.06). The ratio of men to women was 20/10 in the glioma group and 11/10 in the control group. There was no significant difference in BMI between the two groups (median BMI: glioma: 25; control: 28; *p* = 0.05). Arterial hypertension was found in 17 patients in the glioma group and in 6 patients in the control group (*p* = 0.08). Diabetes mellitus was diagnosed in 3 patients in the glioma group and in 2 patients in the control group (*p* > 0.99). The characteristics of the patients in the overall cohort are summarized in Table 2.

**Table 2:** Patient characteristics of the studied cohort

	Glioma (*N* = 30)	Control (*N* = 21)	*p* value
Age in years (median ± SD)	62 ± 3.1	50 ± 4.3	0.06
Sex (M/F)	20/10	11/10	0.3
BMI (kg/m^2^) (median ± SD)	26 ± 0.7	30 ± 1.8	0.05
Comorbidities			
Hypertension	17	6	0.08
Diabetes mellitus	3	2	> 0.99
Post-stroke	3	0	0.26

*BMI* body mass index

### CSF-metabolome profile of all glioma patients compared to controls

**Fig. 1:**
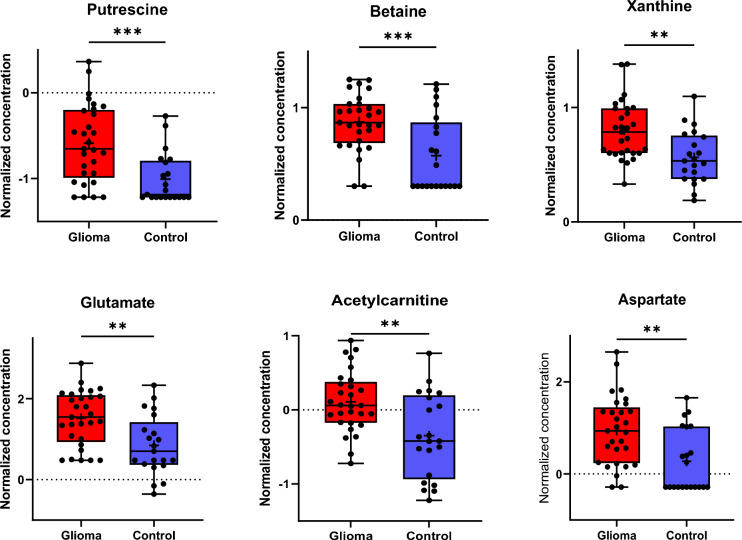
Illustration of the six CSF metabolites that showed the most significant differences between the glioma and control groups according to the FDR-corrected *t* test. The graphs show normalized data (log10). Due to this normalization process, negative values were obtained for some metabolites. Values below the LOD were replaced by the reference value provided by the manufacturer (LOD/2)

**Fig. 2:**
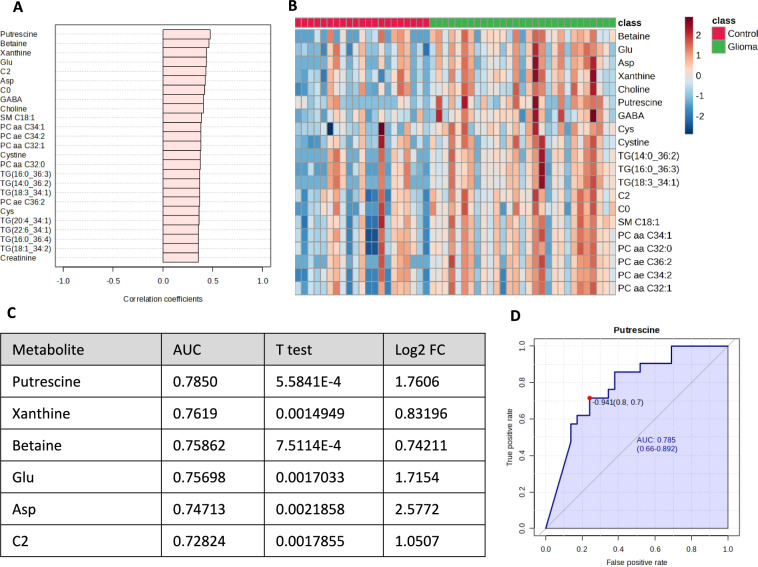
Comparison of the CSF-metabolome profiles of glioma patients and control patients. **A** The top 25 metabolites correlated with glioma vs. control (Spearman rank correlation). The light red bars indicate positive correlations. **B** Heatmap of the top 20 metabolites (*t* test, using Euclidean clustering, Ward method). Each colored cell on the map corresponds to a concentration, with samples in rows and compounds in columns. Red and blue indicate higher or lower metabolite concentrations, respectively. The numbers of patients in the glioma group corresponding to Table 1 from left to right are as follows: 4, 5, 6, 7, 8, 9, 10, 11, 12, 14, 22, 16, 17, 3, 1, 18, 20, 19, 21, 22, 23, 24, 25, 26, 27, 28, 29, 2, and 13. **C** Table showing the univariate ROC analysis of the metabolites selected according to the volcano plot. Putrescine, xanthine, betaine, glutamate, aspartate, and acetylcarnitine (C2) showed considerable areas under the curve (> 0.7). **D** Example ROC curve of putrescine with an AUC of 0.785. *Glu* Glutamate, *Asp* aspartate, *C0* carnitine, *GABA* γ-aminobutyric acid, *Cys* cysteine

Of the 131 out of 630 detectable metabolites in CSF that were suitable for analysis, six showed a significant difference in concentration in both groups according to the FDR-corrected *t* test, as shown in Fig. 1. Compared with that in the control group, the concentration of putrescine significantly increased in the glioma group, with the most significant difference found for putrescine (*p* value of 0.00005). According to the correlation analysis, several other metabolites showed increased CSF concentrations in the glioma group, but the difference was not significant (Fig. 2A). Likewise, the heatmap that shows the relative concentrations of the top 20 metabolites for each patient illustrated that almost all significantly different metabolites were increased in concentration in most samples in the glioma group compared to those in the control group. The numeration of the glioma group in the heatmap (see figure legend) corresponds to the patients’ numbers in Table 1 (Fig. 2B). By applying ROC curve analysis for the identification of biomarker candidates, all six metabolites that were determined by *t* tests (Fig. 1) exhibited an area under the curve (AUC) > 0.7 (Fig. 2C). Again, putrescine also had the most notable effect, with an AUC of 0.785 (Fig. 2D). Among the 51 samples (30 glioma and 21 control) and 131 metabolites, 8 had missing values, which were managed as described previously 2.2.

### CSF-metabolome profile of GBM subgroup compared to controls

**Fig. 3:**
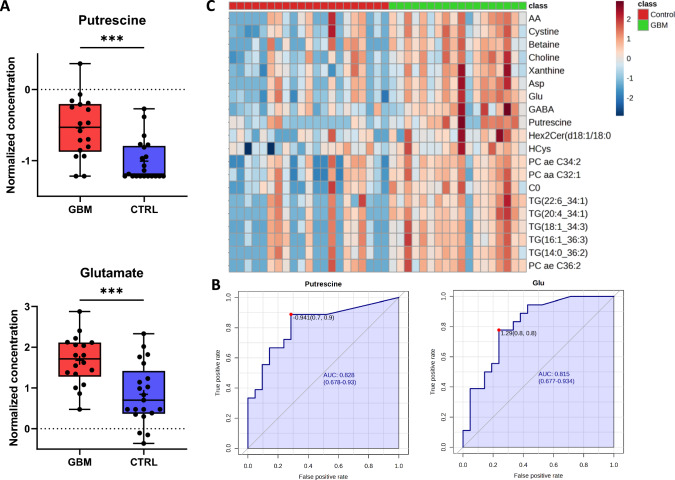
Comparison of the CSF-metabolome profiles of patients with primary GBM and control patients. **A** Normalized concentrations of putrescine and glutamate in CSF of GBM patients vs. controls. **B** Example ROC curve of putrescine with an area under the curve (AUC) of 0.828 and glutamate with an AUC of 0.815. **C** Heatmap of the top 20 metabolites (*t* test, using Euclidean clustering, ward method). Each colored cell on the map corresponds to a concentration, with samples in rows and compounds in columns. Red and blue indicate higher or lower metabolite concentrations, respectively. The numbers of patients in the GBM group correspond to Table 1 from left to right are as follows: 4, 6, 7, 8, 10, 12, 17, 3, 18, 20, 21, 22, 23, 25, 27, 28, 29, 2. *AA* arachidonic acid, *Asp* aspartate, *Glu* glutamate, *GABA* γ-aminobutyric acid, *C0* carnitine, *HCys* homocysteine, *PC* phosphatidylcholine, *TG* triglyceride, *HexCer* hexosylceramide

The complementary comparison of the CSF-metabolome profiles of control patients with those of patients with primary glioblastoma (*n* = 18) revealed significant differences. Out of 630 detectable metabolites in the CSF, 131 were suitable for analysis, and 88 showed a significant difference in concentration between the two groups according to the FDR-corrected *t* test. The metabolites with the highest significance (according to *p* value) were putrescine, cystine, glutamate, aspartate, xanthine, and p-cresol-SO4. In the ROC curve analysis, all six metabolites exhibited an area under the curve (AUC) > 0.7. Figure 3A, B shows the concentration differences and the AUC for the two most relevant metabolites, putrescine and glutamate. Figure 3C represents the heat map of the top 20 metabolites with the largest differences between the GBM and control patients.

The FDR-corrected *T* test showed no differences between the CSF-metabolomic profiles of all patients with primary glioma compared to the group of patients with glioma recurrence (Supplemental Figure S1). The same applied to the comparison of the CSF-metabolomic profiles of primary glioblastoma and glioblastoma recurrence (Supplemental Figure S2).

### Comparison of the serum metabolome profiles of glioma patients and controls

**Fig. 4:**
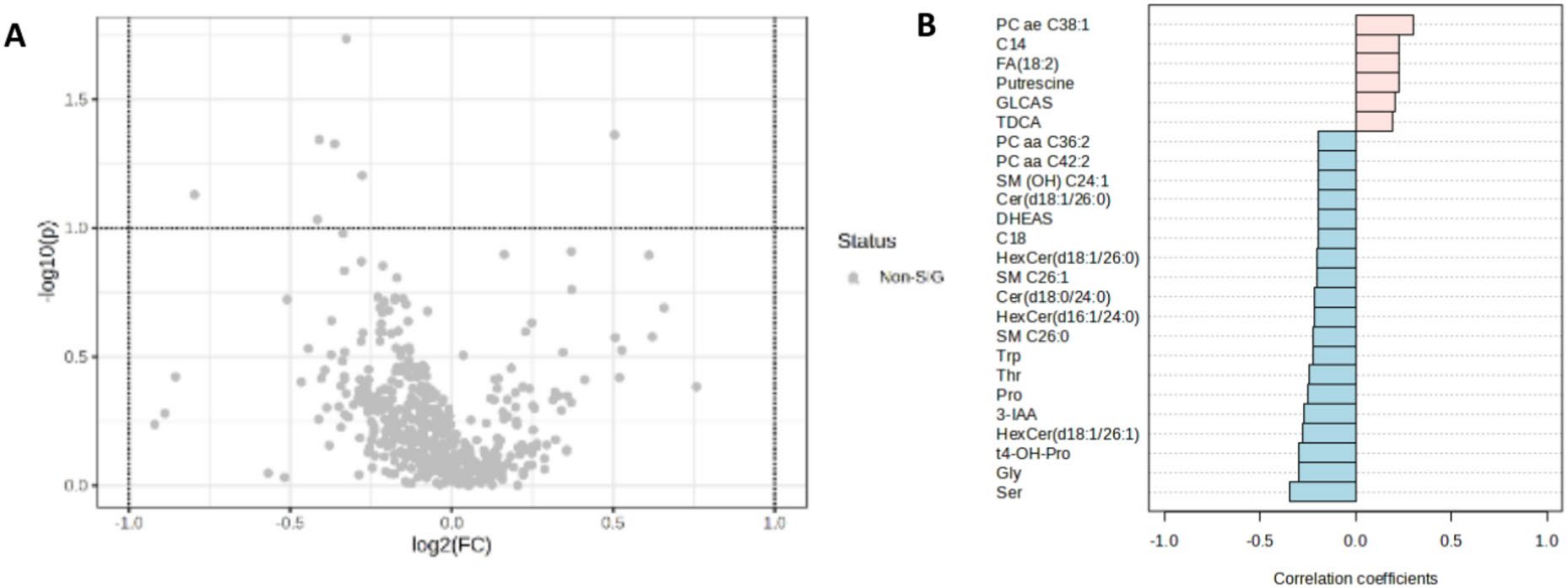
Comparison of the serum metabolome profiles of glioma patients and control patients. **A** Volcano plot showing no significant differences for the investigated metabolites. Each point represents a metabolite. **B** Plot of correlation coefficients of the top 25 metabolites and their relative concentrations between the two groups. Metabolites with red bars are upregulated in the glioma group compared to the control group, and metabolites with blue bars are downregulated. *C14* Tetradecanoylcarnitine, *FA 18:2* octadecadienoic acid, *GLCAS* glycolithocholic acid sulfate, *TDCA* taurodeoxycholic acid, *DHEAS* dehydroepiandrosterone sulfate, *C18* octadecanoylcarnitine, *Trp* tryptophan, *Thr* threonine, *Pro* proline, *3-IAA* 3-indoleacetic acid, *t4-OH-Pro* trans-4-hydroxyproline, *Gly* glycine, *Ser* serine

Serum metabolome profiling revealed no metabolites with significant concentration differences between the two groups (Fig. 4A). In trend, few metabolites, including putrescine, showed increased concentrations in the serum of glioma patients, whereas the majority of metabolites in the serum were reduced (Fig. 4B). There were 506 out of 630 metabolites detectable > LOD in more than 80% of the samples in at least one group. No values were missing.

However, if only the serum metabolome profile of patients with primary GBM was considered in comparison to control patients, significant differences were found. Of the 506 out of 630 detectable metabolites in the serum that were suitable for analysis, cortisol and DHEAS showed a significant higher concentration in the primary GBM group according to the FDR-corrected *t* test (Supplemental Figure S3).

### CSF-metabolome profile of IDH-mutant glioma compared to wild-type glioma

**Fig. 5:**
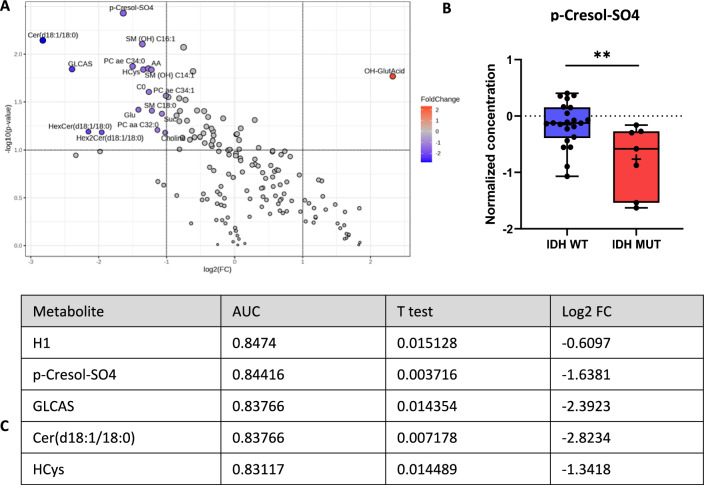
CSF-metabolome profile of patients with IDH-mutant gliomas (*n* = 7) compared to patients with wild-type gliomas (*n* = 22). **A** Volcano plot. Each point represents a metabolite; red (1/160) represents upregulated metabolites in IDH-mutant glioma compared with IDH wild-type glioma, blue (17/160) represents downregulated metabolites, and gray represents metabolites with no difference between the IDH-mutant and IDH wild-type glioma groups [raw *p* value < 0.05 and fold change (FC) > 2]. **B** Representative representation of the metabolite p-cresol sulfate (p-Cresol-SO4), which showed the most significant difference in concentration in the group comparison and was significantly increased in the glioma wild-type group (FDR-corrected *t* test). **C** According to the univariate ROC curve analysis, among the metabolites selected according to the volcano plot, hexoses (H1), p-cresol sulfate (p-cresol-SO4), glycolithocholic acid sulfate (GLCAS), ceramide (d18:1/18:0), and homocysteine (HCys) had considerable AUCs (> 0.83). *AA* arachidonic acid, *C0* carnitine, *Glu* glutamate, *HexCer* hexosylceramides, *OH-GlutAcid* 3-Hydroxyglutaric acid, PC aa/ae glycerophospholipids, *SM (OH)* sphingolipids, *SM* sphingomyelin, *Suc* succinic acid, *WT* wild type

When comparing the CSF-metabolome profiles of patients with IDH-mutated glioma and those with wild-type glioma, 18 metabolites showed considerable differences in concentration according to the volcano plot (FC and raw *p* value) (Fig. 5A). These differences were not reflected by the FDR-corrected t test, as the set limits were not met.

3-Hydroxyglutaric acid (OH-GlutAcid) was significantly increased in the CSF of patients with IDH-mutated glioma, whereas the other 17 metabolites, especially p-cresol sulfate (p-cresol-SO4), homocysteine (HCys), glycolithocholic acid sulfate (GLCAS), hexoses (H1), and ceramide (d18:1/18:0), were decreased. P-Crescol-SO4 showed the most significant difference in concentration (Fig. 5B, C) and proved to be a potentially suitable biomarker in the ROC analysis, with an AUC of 0.8442.

For this analysis, 160/630 metabolites were selected as described previously in 2.2. Among 29 samples (22 IDH WT, 7 IDH MUT) and 160 compounds, there were 7 missing values.

### CSF-metabolome profile of patients with a hypermethylated MGMT promoter compared to patients without MGMT promoter hypermethylation

In the CSF of patients with GBM with a methylated MGMT promoter, three triglycerides, TG (20:3_36:3), TG (16:0_38:3), and TG (18:2_36:2), were found to be significantly elevated according to the volcano plot. These differences were not reflected by the FDR-corrected t test, as the set limits were not met. For this analysis, 198/630 metabolites were selected as described in Sect. 2.2. Among 21 (9 with MGMT+ and 12 with MGMT) samples and 180 compounds, 6 had missing values.

## Discussion

Here, we analyzed the serum and CSF-metabolome profiles of 30 glioma patients and compared them with profiles from 21 control patients, thereby contributing to the understanding of metabolomic changes in gliomas and potentially improving diagnostic methods. No significant differences were found in the serum profiles of glioma patients compared to controls, suggesting that serum may not be suitable for this purpose (Fig. 4). It should be mentioned that individual significant metabolites could be detected if only the sera of the glioblastoma patients were considered in comparison to the control group (Supplemental Figure S3). Here, the significantly increased cortisol value was particularly noticeable, which is most likely due to the oral dexamethasone medication required by glioblastoma patients (Table 1) or the increased stress reaction following the usually more complex glioblastoma operation.

In the CSF, 6 out of the 131 detectable metabolites were significantly increased in the glioma group. These included the neurotransmitter glutamate and the biogenic amine putrescine. Among the metabolites detected, putrescine appears to be the most promising biomarker candidate for differentiating between gliomas and non-malignant brain tumors, as indicated by an AUC of 0.785 (Figs. 1, 2). When considering only the glioblastoma patients within the glioma group, similar results were detected regarding the differential CSF-metabolome profile compared to the control cohort (Fig. 3). Putrescine, a metabolic precursor of spermidine and spermine, is primarily synthesized by the enzyme ornithine decarboxylase [12]. Since the 1980s, studies have shown elevated putrescine concentrations and ornithine decarboxylase activity in brain tumors [13, 14], with some indications suggesting a correlation between polyamine levels and tumor malignancy [15]. In 2001, Ernestus and colleagues identified ornithine decarboxylase activity as a marker of brain tumor malignancy; ornithine decarboxylase activity was notably greater in gliomas and increased with tumor severity, although polyamine concentrations did not consistently correlate with the degree of malignancy in their study of 670 patients [16]. Recent research has further explored the role of polyamines in cancer and autoimmunity. Notably, reduced polyamine levels are observed in autoimmune disorders such as systemic lupus erythematosus and are correlated with increased inflammatory responses [17, 18]. This finding is in line with the suggestion that polyamines inhibit antitumor responses in tumors such as malignant gliomas [19]. Antineoplastic therapies targeting polyamine synthesis and uptake have shown promising effects, for instance, reducing tumor size in neuroblastoma xenograft models [19]. Polyamines also influence the immune microenvironment of solid tumors, particularly through the recruitment of immunosuppressive tumor-associated macrophages (TAMs), which express [20–22] the enzyme arginase-1 involved in putrescine production, to CNS tumors [23, 24]. Due to its chemical properties as an amine, it appears to function as a buffering substance, thereby promoting the survival of anti-inflammatory and therefore immunosuppressive tumor-associated myeloid cells [25]. These findings reinforce the significant role of polyamines in tumor biology, especially in CNS malignancies, suggesting that putrescine could serve as both a diagnostic CSF marker and a therapeutic target in malignant glioma [25].

In addition to putrescine, the CSF-metabolome profile analysis of patients with gliomas showed significant differences in the concentrations of aspartate, glutamate, and acylcarnitine compared to those in the control group. The elevated glutamate levels in the glioma group align with the previous findings that glutamate signaling promotes glioma invasion and growth [26]. Glioma cells often release large amounts of glutamate through glutaminase, which converts glutamine to glutamate [27], coupled with potential impairment in extracellular glutamate removal [28]. Consequently, the glutamatergic axis has been considered a possible therapeutic target for glioma treatment, including for the investigation of tumor treatments aimed at disrupting neuroglioma signaling [29]. Aspartate becomes crucial when glucose and glutamine, the primary carbon sources in rapidly proliferating cells, are depleted. High aspartate levels are essential for maintaining key metabolic pathways such as purine/pyrimidine synthesis and the generation of nonessential amino acids [30]. Elevated aspartate concentrations have previously been reported in human glioma tissue metabolomic studies [31].

Moreover, cancer can significantly alter lipid metabolism [32–35]. In 2020, researchers at Ohio State University reported that the enzyme diacylglycerol acyltransferase 1 is upregulated in glioblastoma [36]. Inhibition of this enzyme induced cell apoptosis in glioblastoma cells and reduced tumor growth both in vitro and in vivo. Notably, knocking down diacylglycerol acyltransferase 1 caused a marked increase in acylcarnitines, which transport fatty acids into mitochondria for oxidation and energy production in neuronal energy metabolism [37]. Our analysis revealed significantly elevated concentrations of acylcarnitine in patients with malignant glioma compared to controls, suggesting increased lipid metabolism in glioma patients.

To our knowledge, this study represents the largest cohort of patients with gliomas, including glioblastoma, to whom comprehensive metabolomic analysis was performed. A 2018 study by Ballester and colleagues compared CSF from 23 brain tumor patients, including those with brain metastases from lung cancer or breast cancer, with that from 8 controls [38]. However, their CSF samples were obtained by lumbar puncture or via puncture of an Ommaya reservoir, not intraoperatively, as in our study. They identified 43 metabolites with concentration differences in tumor patients, focusing on glycine, arginine, choline, and nitrogen metabolism alterations [38]. Tricarboxylic acid cycle metabolites, including malic acid and succinate, were elevated in the CSF of CNS tumor patients, especially in those with IDH-mutant gliomas. Our study also revealed elevated acylcarnitine levels in glioma patients’ CSF but only in IDH wild-type tumors [38]. A 2022 publication on diffuse glioma tumor tissue metabolome identified distinct metabolites affected by tumor histology, IDH1 mutation status, or therapy. *IDH1* wild-type gliomas had higher neurotransmitter levels, possibly linked to their poorer prognosis [39]. In our study, CSF glutamate differentiated malignant glioma patients from controls but showed no differences between *IDH*-mutated and *IDH* wild-type gliomas. However, p-cresol sulfate, homocysteine, glycolithocholic acid sulfate, ceramide (d18:1/18:0), and hexoses were significantly elevated in the CSF of patients with *IDH* wild-type gliomas (Fig. 5). Only 3-hydroxyglutaric acid was increased in *IDH*-mutated gliomas. p-Cresol sulfate is a sulfate conjugate of the bacterial metabolite p-cresol, which is a uremic toxin and originates when gut bacteria ferment proteins in the large intestine. While there is a well-established link between high blood levels of p-cresol sulfate and mortality from cardiovascular disease [40], a positive correlation of this metabolite with the development of tumor diseases could only be drawn for clear cell renal cell carcinoma [41]. The role of p-cresol sulfate in neurological and neuro-oncological tumor diseases is still unclear, although gut microbiome connections are suspected [42]. The decrease in homocysteine in *IDH1*-mutated glioma patients might be due to a shift toward glutathione synthesis in the hypoxic tumor environment to counterbalance reactive oxygen species [43].

**Fig. 6:**
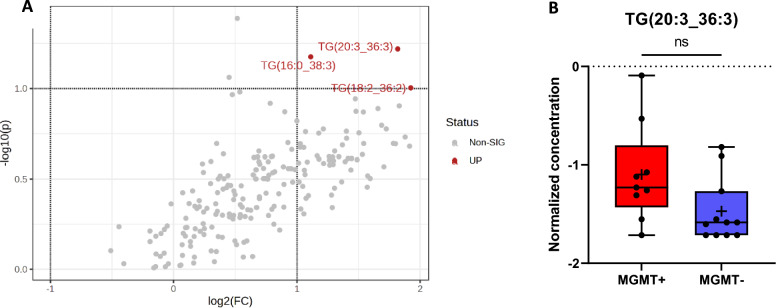
CSF-metabolome profile of glioma patients with a hypermethylated MGMT promoter (MGMT+) (*n* = 9) compared to patients without MGMT promoter hypermethylation (MGMT-) (*n* = 12). **A** Volcano plot. Each point represents a metabolite, red points (3/131) represent upregulated metabolites in the MGMT+ group compared with the MGMT– group, and gray points represent metabolites with no difference between the MGMT+ and MGMT– groups [raw *p* value < 0.1 and fold change (FC) > 2]. **B** Exemplary illustration of the metabolite TG (20:3_36:3) and comparison of its concentrations using an FDR-corrected *t* test

In our study, the CSF-metabolomic profile of patients with MGMT promoter hypermethylation showed only minor alterations. Specifically, the levels of three triglycerides [TG (20:3_36:3), TG (16:0_38:3), and TG (18:2_36:2)] were significantly greater in patients with MGMT promoter hypermethylation than in patients without *MGMT* promoter hypermethylation (Fig. 6). To date, there has been limited research in this area. However, a recent publication proposed an enrichment of metabolites related to glycerophospholipid metabolism and sphingolipid metabolism, findings that are in line with our results [44].

In addition to the important findings that could influence the diagnostic landscape for gliomas, this study has certain limitations. In particular, the number of patients in the sub-analyses on IDH mutation status and *MGMT* methylation status was limited and the glioma group included both patients with primary glioma and those with tumor recurrence. However, it should be noted that the CSF-metabolome profiles of primary glioma vs. glioma recurrence, as well as those of primary GBM vs. GBM recurrence, did not show any significant differences. In addition to the tumor diagnosis itself, the patients within the glioma cohort differed in terms of tumor location, tumor volume, required steroid dose, extent of tumor necrosis, or possible neurological comorbidities. While tumor localization, neurological comorbidities, or the presence of epileptic seizures likely had less impact in the cohort presented in this study, the influence of oral steroids on the metabolome profile, in particular, must be considered. This likely applies specifically to the differences detected in the serum metabolome profiles of control and GBM patients. A further limitation concerns the control cohort, which was rather heterogeneous. A certain impact of the underlying diseases in the control group on the metabolome profile in the CSF cannot be ruled out. However, at least in patients with vestibular/trigeminal schwannoma—conditions distant from the CSF compartment—or in patients with hydrocephalus or idiopathic intracranial hypertension, a minor influence can be assumed. The same consideration applies to patients with non-tumorous changes such as colloid or arachnoid cysts. In future studies, the metabolome profiles of non-malignant brain tumor diseases, such as meningioma, should be compared with those of malignant tumor diseases in a differentiated manner, if possible.

## Conclusions

This study successfully demonstrated that the CSF-metabolome profile of glioma and especially GBM patients significantly differed from that of controls. In particular, putrescine was identified as a potential diagnostic biomarker for gliomas. In addition, it was possible to correlate the metabolome signature in the CSF with the IDH status within the glioma group. The next steps will involve validating these results in a larger, multicenter cohort and investigating whether differences in subgroups would have an instructive impact on outcome prediction.

## Supplementary Information

Below is the link to the electronic supplementary material.

Supplementary file1 (DOCX 2438 KB)

## Data Availability

The raw data supporting the conclusions of this article will be made available by the authors without undue reservation.

## Abbreviations

3-IAA: 3-Indoleacetic acid
AUC: Area under the curve
BSC: Best supportive care
BMI: Body mass index
C0: Carnitine
C14: Tetradecanoylcarnitine
C18: Octadecanoylcarnitine
CRTx: Chemoradiotherapy
CSF: Cerebrospinal fluid
CTx: Chemotherapy
DHEAS: Dehydroepiandrosterone sulfate
FA 18:2: Octadecadienoic acid
FDR: False discovery rate
F: Female
GABA: γ-Aminobutyric acid
GBM: Glioblastoma
GLCAS: Glycolithocholic acid sulfate
Glu: Glutamate
Gly: Glycine
GTR: Gross total resection
HCys: Homocysteine
HexCer: Hexosylceramides
H1: Hexoses
IDH: Isocitrate dehydrogenase
KPS: Karnofsky performance score
LOD: Limit of detection
M: Male
MGMT: O6-Methylguanine methyltransferase
Mut: mutant
N/A: Not applicable
OH-GlutAcid: 3-Hydroxyglutaric acid
p-Cresol-SO4: p-Cresol sulfate
Pro: Proline
ROC: Receiver operating characteristic
RTx: Radiotherapy
Ser: Serine
SM (OH): Sphingolipids (Hydroxylated)
SM: Sphingomyelin
STB: Stereotactic biopsy
STR: Subtotal resection
Suc: Succinic acid
TAMs: Tumor-associated macrophages
TDCA: Taurodeoxycholic acid
TG: Triglyceride
Thr: Threonine
Trp: Tryptophan
TTF: Tumor-treating fields
t4-OH-Pro: Trans-4-hydroxyproline
WT: Wild type
