# Correction: Metabolomic profile of cerebrospinal fluid from patients with diffuse gliomas

**DOI:** 10.1007/s00415-024-12722-5

**Published:** 2024-11-19

**Authors:** Nora Möhn, Harold F. Hounchonou, Sandra Nay, Philipp Schwenkenbecher, Lea Grote-Levi, Fadi Al-Tarawni, Majid Esmaeilzadeh, Sven Schuchardt, Kerstin Schwabe, Herbert Hildebrandt, Hauke Thiesler, Friedrich Feuerhake, Christian Hartmann, Thomas Skripuletz, Joachim K. Krauss

**Affiliations:** 1 Department of Neurology, Hannover Medical School, Carl-Neuberg-Straße 1, 30625 Hannover, Germany; 2 Department of Neurosurgery, Hannover Medical School, Hannover, Germany; 3 Fraunhofer Institute of Toxicology and Experimental Medicine, Hannover, Germany; 4 Institute of Clinical Biochemistry, Hannover Medical School, Hannover, Germany; 5 Department of Neuropathology, Institute of Pathology, Hannover Medical School, Hannover, Germany

**Correction: Journal of Neurology (2024) 271:6970–6982** 10.1007/s00415-024-12667-9

In the original version of this article, the author’s name Majid Esmaeilzadeh was incorrectly written as Majid Esmailezadeh.

The original article has been corrected.

