# Supplementary material for: Metabolomic profile of cerebrospinal fluid from patients with diffuse gliomas
Source: J Neurol. 2024 Sep 3;271(10):6970–82. doi: 10.1007/s00415-024-12667-9 (PMC11446983; doi:10.1007/s00415-024-12667-9)
Supplement: Supplementary file 1 — Supplementary file1 (DOCX 2438 KB) [file 415_2024_12667_MOESM1_ESM.docx]

**Supplementary material**

| **ID** | **Sex** | **Age (years)** | **Diagnosis** |
| --- | --- | --- | --- |
| 1 | F | 82 | Vestibular schwannoma |
| 2 | M | 52 | Vestibular schwannoma |
| 3 | M | 61 | Amyloidangiopathy |
| 4 | F | 49 | Arachnoid cyst |
| 5 | M | 75 | Cerebral infection |
| 6 | M | 17 | Hydrocephalus |
| 7 | F | 34 | Hypophysenadenom |
| 8 | F | 78 | Hydrocephalus |
| 9 | F | 65 | Cavernous malformation |
| 10 | F | 39 | Colloid cyst |
| 11 | F | 63 | Meningioma |
| 12 | M | 67 | Cerebral metastasis |
| 13 | M | 63 | Cerebral metastasis |
| 14 | M | 26 | Neurocytoma |
| 15 | M | 58 | Meningioma |
| 16 | M | 56 | Meningioma |
| 17 | F | 32 | Idiopathic intracranial hypertension |
| 18 | F | 23 | Idiopathic intracranial hypertension |
| 19 | F | 29 | Idiopathic intracranial hypertension |
| 20 | M | 61 | Hydrocephalus |
| 21 | F | 26 | Trigeminal schwannoma |

**Supplementary Table 1 (Table S1):** Patient characteristics of the control group.


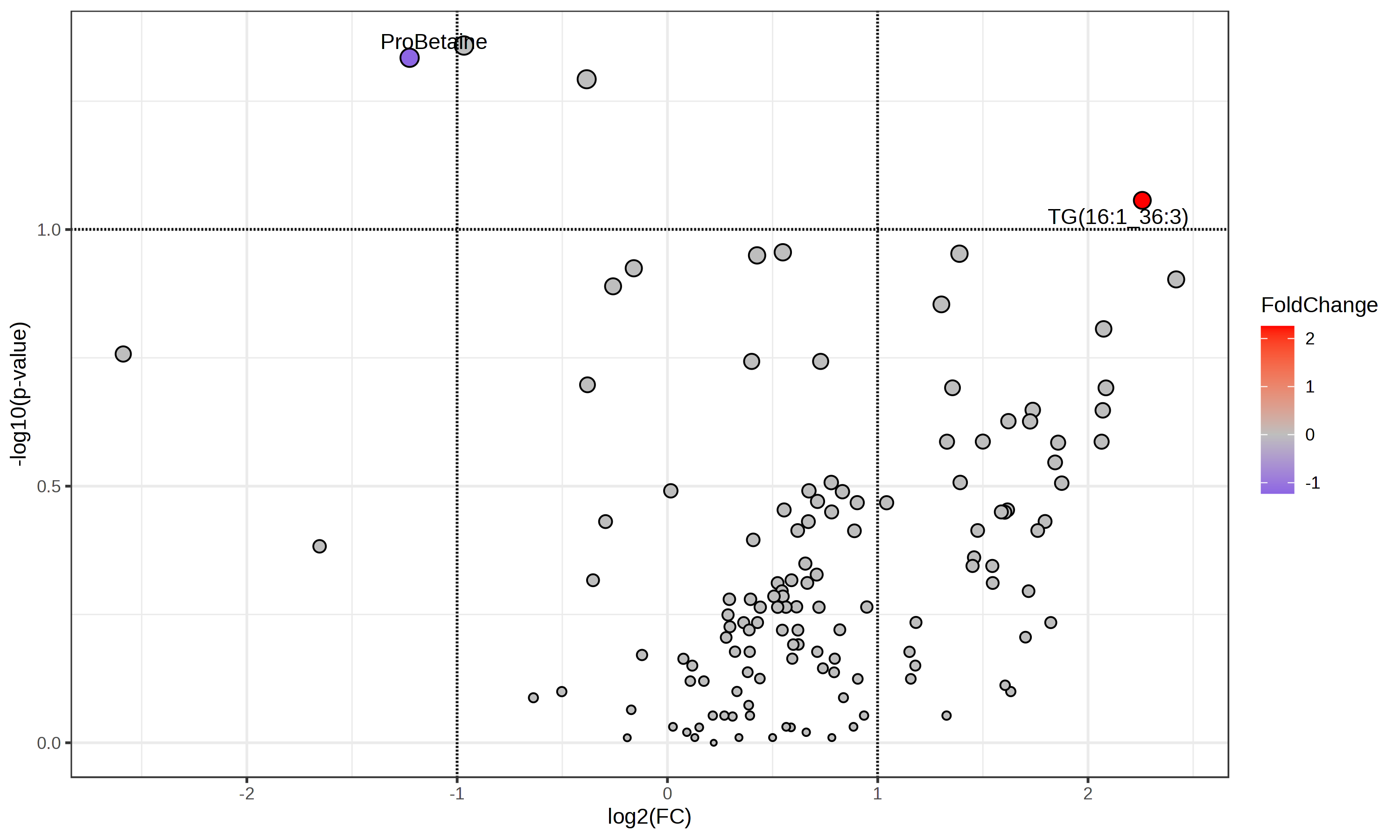


**Supplemental Figure S1:** Comparison of the CSF metabolome profiles of patients with primary glioma and patients with glioma recurrence. Volcano plot showing significant differences in concentrations of pro-betaine and TG(16:1_36:3). However, the FDR corrected T test showed no differences between the CSF-metabolomic profiles of both patient groups.


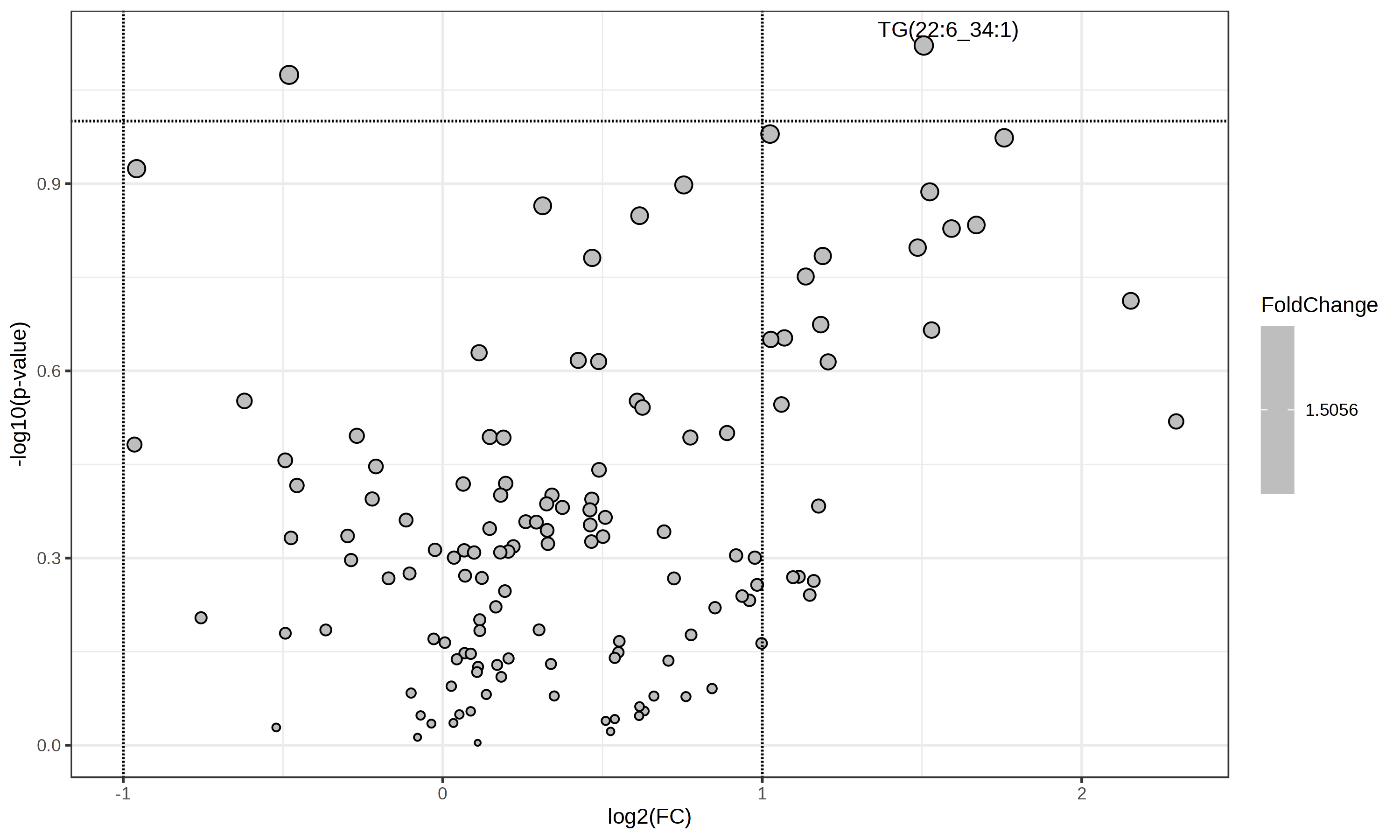


**Supplemental Figure S2:** Comparison of the CSF metabolome profiles of patients with primary glioblastoma and patients with glioblastoma recurrence. Volcano plot as well as FDR corrected T test showed no significant differences between the two patient groups.


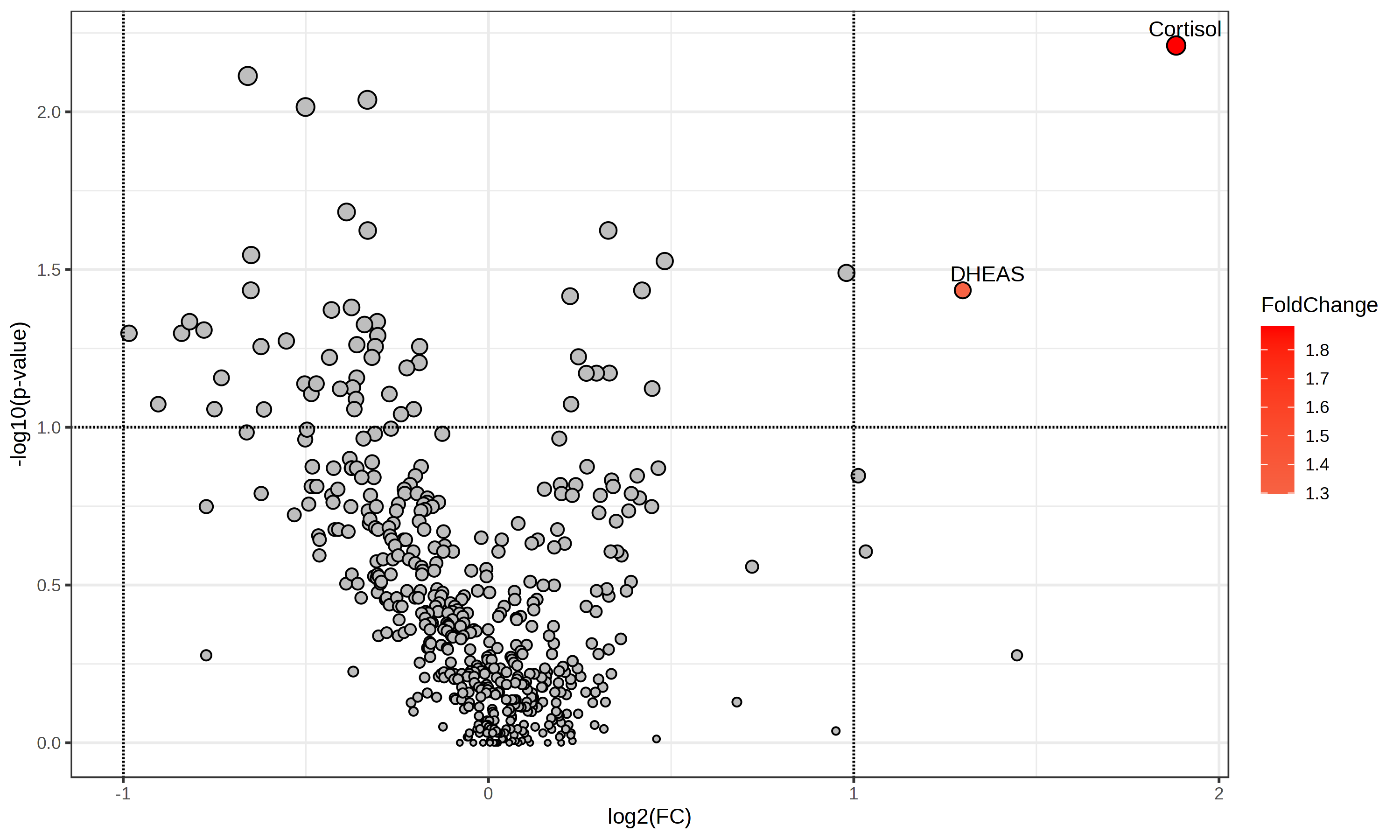


**Supplemental Figure S3:** Comparison of the serum metabolome profiles of patients with primary GBM and control patients. Volcano plot showing significant differences in concentrations of cortisol and dehydroepiandrosterone sulfate (DHEAS). Patients within the GBM group exhibited significantly higher serum-concentrations of both metabolites. Each point represents a metabolite.
